# Modification of Sunlight Radiation through Colored Photo-Selective Nets Affects Anthocyanin Profile in *Vaccinium* spp. Berries

**DOI:** 10.1371/journal.pone.0135935

**Published:** 2015-08-19

**Authors:** Laura Zoratti, Laura Jaakola, Hely Häggman, Lara Giongo

**Affiliations:** 1 Genetics and Physiology Unit, University of Oulu, Oulu, Finland; 2 Climate laboratory, Department of Arctic and Marine Biology, UiT The Arctic University of Norway, Tromsø, Norway; 3 Norwegian Institute of Bioeconomy Research, NIBIO Holt, Tromsø, Norway; 4 Genomics and Biology of Fruit Crop Department, Research and Innovation Center, Edmund Mach Foundation, S. Michele all'Adige, Trento, Italy; Zhejiang University, CHINA

## Abstract

**Objectives:**

In recent years, the interest on the effects of the specific wavelengths of the light spectrum on growth and metabolism of plants has been increasing markedly. The present study covers the effect of modified sunlight conditions on the accumulation of anthocyanin pigments in two *Vaccinium* species: the European wild bilberry (*V*. *myrtillus* L.) and the cultivated highbush blueberry (*V*. *corymbosum* L.).

**Methods:**

The two *Vaccinium* species were grown in the same test field in the Alps of Trentino (Northern Italy) under modified light environment. The modification of sunlight radiation was carried out in field, through the use of colored photo-selective nets throughout the berry ripening during two consecutive growing seasons. The anthocyanin profile was then assessed in berries at ripeness.

**Results:**

The results indicated that the light responses of the two *Vaccinium* species studied were different. Although both studied species are shade-adapted plants, 90% shading of sunlight radiation was beneficial only for bilberry plants, which accumulated the highest content of anthocyanins in both seasons. The same condition, instead, was not favorable for blueberries, whose maturation was delayed for at least two weeks, and anthocyanin accumulation was significantly decreased compared to berries grown under sunlight conditions. Moreover, the growing season had strong influence on the anthocyanin accumulation in both species, in relation to temperature flow and sunlight spectra composition during the berry ripening period.

**Conclusions:**

Our results suggest that the use of colored photo-selective nets may be a complementary agricultural practice for cultivation of *Vaccinium* species. However, further studies are needed to analyze the effect of the light spectra modifications to other nutritional properties, and to elucidate the molecular mechanisms behind the detected differences between the two relative *Vaccinium* species.

## Introduction

Sunlight radiation and its spectral composition regulate many physiological responses in plants, including flavonoid biosynthesis. In the last few years, the effect of spectral wavelengths on the accumulation of flavonoids in fruit species has been investigated, as reviewed recently [1]. High solar intensities and predominance of shorter wavelengths in the light spectrum, in the range of blue and UV light wavelengths, generally increase the amount of flavonoids, and in particular the class of anthocyanins (ACs), during ripening, harvesting and post-harvesting of fruits.

Among berry fruits, *Vaccinium* spp. are considered one of the richest sources of flavonoids, and are recognized for their high antioxidant activity and benefits on brain, liver and stomach health [2–4]. *Vaccinium* spp. comprise the cultivated blueberries, e.g. the Northern and Southern highbush blueberries, but also the wild lowbush species [5]. There is significant variation in the flavonoid content and antioxidant activity between the fruits of blueberry varieties and within other *Vaccinium* species [6].

The content of flavonoids is affected by genetic differences and also by physiological processes which take place during maturation and ripening of berry fruits. Many complex biochemical changes occur within the fruit during this stage, resulting in softening, sweetening, cell enlargement and pigmentation [7]. At the early stage of maturation, when fruit is green, chlorophylls, carotenoids and flavonoids, e.g. flavonols, are the predominant fruit pigments, while color changes during the maturation—when the berry turns from green to pink, as chlorophylls are degraded and ACs start to accumulate [5, 8]. The synthesis of ACs in the berries of both wild and cultivated species correlates with the expression of the flavonoid pathway genes during the fruit ripening (e.g. bilberry [9] and blueberry [10, 11]). However, in the highbush blueberry, ACs are mainly present in the berry skin and outer layer of the pulp, while in bilberries ACs accumulate also in the fruit flesh [12].

AC accumulation is also highly influenced by the environment. Generally, ACs accumulate in plant leaves, flowers and fruits [12]. The light environment plays an important role in regulation of AC accumulation in fruit tissues, as photoperiod, intensity of the radiation and composition in wavelengths of the light spectrum influence the expression of genes along the flavonoid pathway and related transcription factors [1]. The light quality has been shown to affect the biosynthesis of flavonoids during the ripening of berries for instance in grapevine (*Vitis* spp.) [13–15], strawberry (*Fragaria x ananassa*) [16, 17] and *Vaccinium* spp. [17–20]. Another major environmental factor altering AC composition in berries is temperature. High temperatures inhibit AC accumulation, whereas lower temperatures enhance expression of genes in phenylpropanoid pathway as well as AC accumulation [21–23].

In the present study, we compared the accumulation of ACs in two *Vaccinium* species: the European wild bilberry (*V*. *myrtillus* L.) and the cultivated highbush blueberry (*V*. *corymbosum* L.), in response to modified intensity and spectra of sunlight radiation. The modifications were carried out in field through the use of colored photo-selective nets, which are generally used for the management of a wide range of crops, because of their ability to diminish solar radiation and temperature [24], thus optimizing the agronomic performance of the productions. Photo-selective nets have shown to positively affect fruit yield and quality of a shade-adapted plant such as blueberry [25] or to delay the harvest date, which could be economically important to growers [24]. Nevertheless, the effect of the photo-selective nets on the nutritional properties of the berries, such as AC profile, has not to our knowledge studied with *Vaccinium* species so far.

However, our earlier study indicated that the application of monochromatic light during the early berry development increases the accumulation of ACs, affecting the AC profile of bilberries in controlled conditions [20]. Recent studies on other berry species, such as grape [15] and strawberry [16] support the importance of the blue irradiance spectra in the AC biosynthesis. Thus, the aim of the study was to test the effect of increased proportion of blue wavelengths in the sunlight spectra in relation to AC accumulation in *Vaccinium* berries. The experiment was conducted in field, with the application of colored photo-selective nets, in order to also study the interaction of light conditions with other environmental variables, e.g. light intensity and temperature.

## Materials and Methods

### Plant materials

The trial was conducted at Trentino region, Italy (46.07°N, 11.23°E, altitude 548 m above sea level—a.s.l.) on wild and cultivated *Vaccinium* species. The wild bilberry (*V*. *myrtillus* L.) plants were collected from two locations of Trentino (Table 1). Permissions for field work were granted by the municipalities of Grumes, Valda and Zambana and the Province of Trento (Italy). Plants were collected after pollination, when holding immature green fruits and well developed root system, and were placed in plastic boxes (30 x 15 cm) with acid peat soil. The cultivated blueberry (*V*. *corymbosum* L., cv Brigitta Blue) plants were 12 years old and were grown in 50 L pots. This cultivar was chosen as the development of fruits was contemporary with the fruit ripening of bilberry. All plants were fertigated with the same nutritional solution, which included macroelements (NO_3_ 6.6 mmol/L, NH_4_ 4.8 mmol/L, PO_4_ 1.8 mmol/L, K 4.6 mmol/L, Mg 2.5 mmol/L, and Ca 2.2 mmol/L) and microelements (Fe 20 μmol/L, Mn 20 μmol/L, Zn 8 μmol/L, Cu 1.75 μmol/L, B 12 μmol/L and Mo 0.75 μmol/L). The irrigation timing, fertilizer concentration and pH (maintained at 4.5) were all controlled automatically.

**Table 1:** Locations where bilberry plants where collected and later on cultivated in the test field.

Location	Species	Latitude (°N)	Longitude (°E)	Altitude (m a.s.l.)
Val di Cembra 1	*V*. *myrtillus*	46.22	11.26	1261
Val di Cembra 2	*V*. *myrtillus*	46.22	11.24	1404
Vigalzano (common cultivation test field)	*V*. *myrtillus*, *V*. *corymbosum* cv. Brigitta Blue	46.07	11.23	458

Blueberry bushes (cv. Brigitta Blue) were already grown in the test field.

### Experiment setting with colored photo-selective nets

Plants were placed under Blue, Pearl (below called ‘white’) and Red IRIDIUM nets from Agritenax (Eboli, Salerno, Italy) when fruits were at immature green stage. The nets had 15%, 7% and 9% shading according to the company specifications, therefore white and red nets were doubled to uniform as much as possible the shading among treatments to 25%. In case of red net, doubling the layers of nets over the crops helped to cut away the blue bands, thus increasing the proportion of red light reaching the plants. The effect of the nets was compared with plants grown under sunlight–in open-field without any screen. Almost complete light exclusion conditions were also tested using a black net which induced a 90% shading of sunlight. Nets were arranged in blocks of 10 m x 10 m and 2.5 m high.

**Fig 1:**
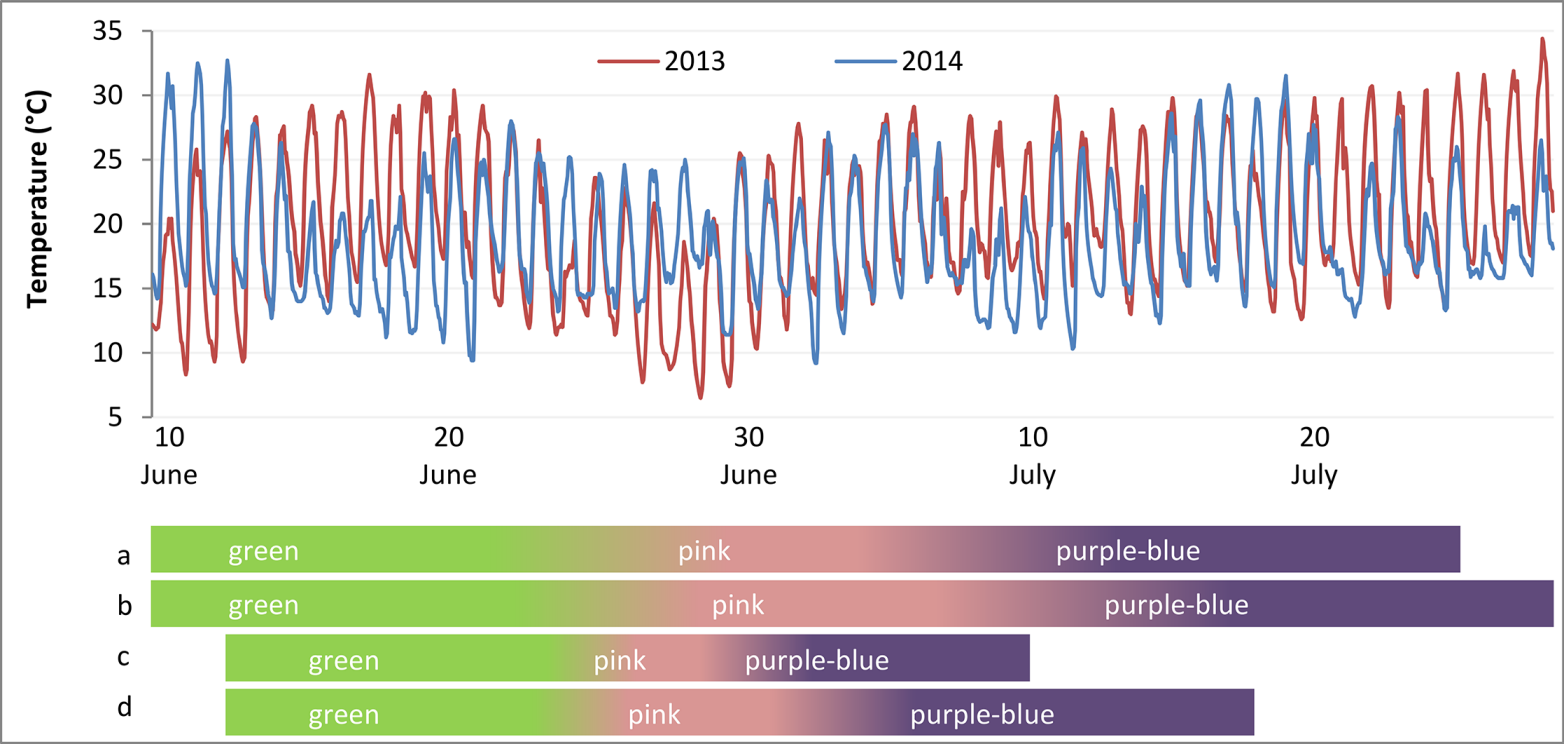
Daily temperatures measured during the ripening period of *Vaccinium* berries in two consecutive seasons (2013–2014). Colored bars show the ripening process, through green, pink and purple-blue (fully ripe) stages of bilberries in 2013 (a), in 2014 (c) and blueberries in 2013 (b), in 2014 (d).

Bilberry plants (a minimum of two per origin–Table 1 –for at least three biological replicates under each treatment) were placed under the nets when berries were at immature green stage (stage 2, [20]) on June 10, 2013. Berries were picked on July 25, 2013 when fully colored (stage 6, [20]) and total soluble solid content (TSSC, see below) was higher than 8.0°Brix. The same was repeated the following season, when plants were placed under the nets on June 13, 2014 and picked between July 3 and 10, 2014 (the ripening process is presented in Fig 1).

Similarly, three blueberry plants were transferred under each treatment when berries were at immature green stage (stage 2, [26]) on June 4, 2013. The harvesting started on July 16, 2013, when berries were fully colored (stage 7, [26]) and TSSC was higher than 10.0°Brix. Nets were left on the plants until leaves were fallen down, and replaced the following season when berries were again at stage 2 (May 12, 2014) and harvesting started on July 18, 2014 (Fig 1).

After collection, the berries for AC, TSSC and acidity analyses were immediately packed on ice and transferred within 30 minutes to -20°C until analyses. The size and weight of fresh berries were recorded immediately after the harvesting.

### Temperature, humidity and light conditions under photo-selective nets

**Fig 2:**
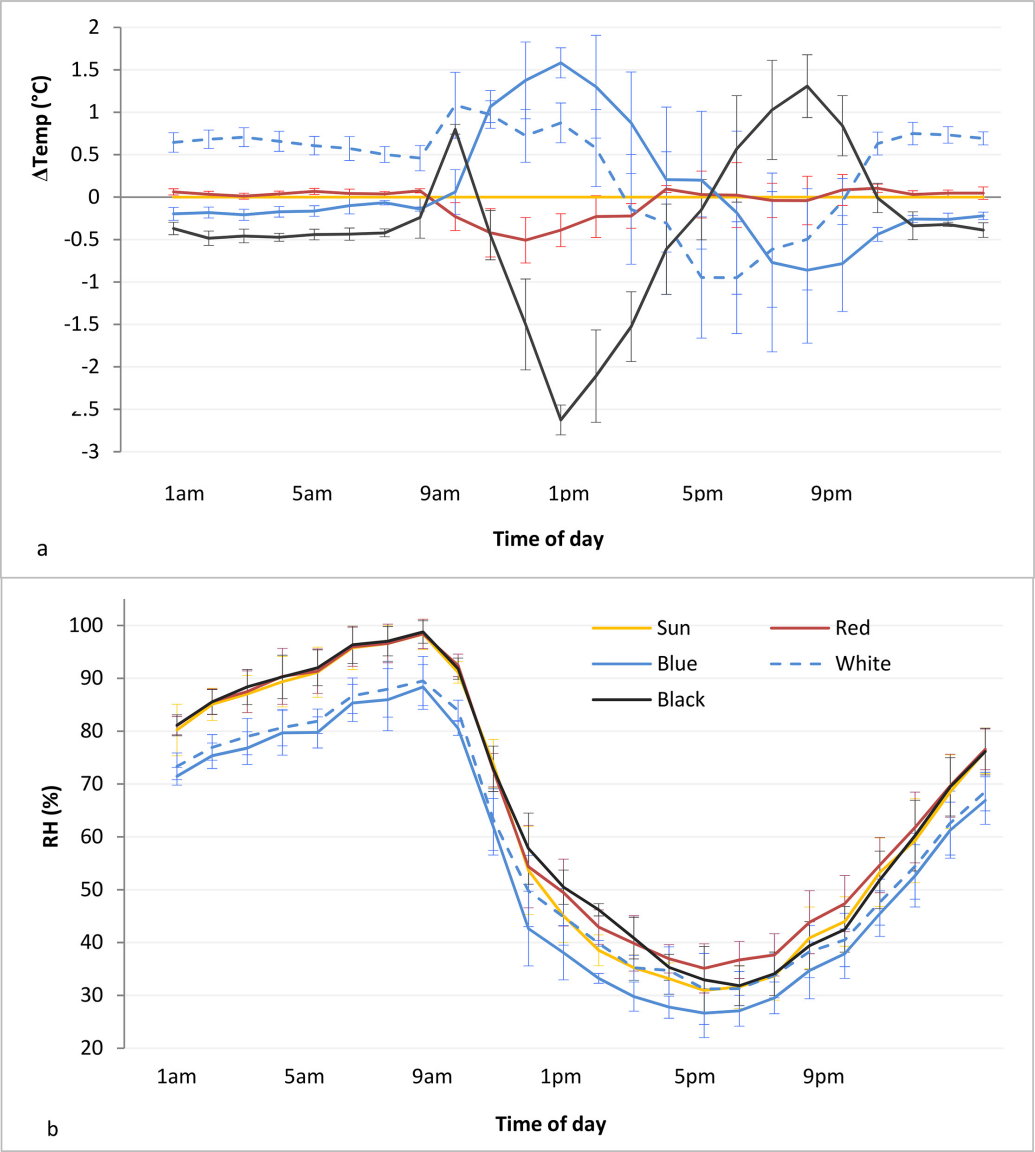
Temperature (a) and relative humidity (RH; b) measured under colored photo-selective nets. Data were recorded every hour continuously for one week during the fruit ripening. Temperature is expressed as difference between temperatures measured under net and under direct sunlight in open-field.

The air temperature and humidity in open-field and under the nets were continuously monitored utilizing the data of the meteorological station in the test field (Table 1) and also by data loggers (Tinytag Plus 2, Gemini Data Loggers Ltd, Chichester, UK; Fig 2). Data loggers were placed 2 m above the ground, protected from direct solar radiation, rain and sprays.

Light was measured with a USB 2000 Spectrometer (Ocean Optics, USA). Under each net, measurements were taken at the top of the canopy every 2^nd^ hour between 9am and 3pm. Measurements were recorded on sunny days under clear sky during the berry ripening. The quality of light reaching the plants was measured as photosynthetic active radiation (PAR) by integrating the area between 200–850 nm for full light spectra, 280–320 nm for UVB, 320–380 nm for UVA, 380–495 nm for blue (B), 495–590 nm for green (G), 590–710 nm for red (R) and 710–750 nm for far-red (FR) [27]. The ratios of B/R and R/FR were calculated. Spectra and integration of light were measured from five consecutive scans of the sunlight, after calibration of the instrument according to the manufacturer’s instructions.

### Photosynthesis

The photosynthetic activity of blueberry plants was analyzed on three different leaves at the top, middle and bottom positions of the bush. PAR and photosynthetic rate were measured in a clear sky day with an infrared gas analyzer (Ciras, PP Systems International, USA).

### Size, weight and TSSC of berries

For each blueberry plant, 20 ripe berries were picked for size and weight analyses. Diameter and height were measured with a digital caliper, and weight of each single berry recorded. Due to the soft nature of the bilberries at ripeness, only TSSC and acidity were measured during the study. The pomological analyses at maturity were conducted on fruits as homogenous as possible for size and color, harvested at stage 7 [26]. At mature stage, TSSC and acidity content were measured with a DBR35 refractometer and a titration Compact Titrator (Crison, Modena, Italy) and with 0.1 N NaOH of 5 g of berry, respectively. Data were expressed in milliequivalents (meq)/100g fresh weight (FW).

### Anthocyanin analyses

Frozen ripe berries were ground into a fine powder with a cryomill (Retsch, Haan, Germany). The grounded material of each sample (1 g FW, out of at least 10 g of sample) was extracted as described in our previous work [20]. Three new AC compounds were identified by screening tandem mass spectrometry (MS/MS) transitions. Chromatography, mass spectrometry conditions and multiple reaction monitoring (MRM) transitions relative to new compounds are described in S1 Table.

### Statistical analyses

The effect of year and different microclimate conditions on the accumulation of ACs in berry fruits was analyzed by two-way ANOVA. The Pearson’s test was conducted to correlate the pomological analyses with the AC content of blueberries. All statistical analyses were handled with STATISTICA 9 software (StatSoft Inc., Tulsa, USA).

## Results

### Berry development and effect of colored photo-selective nets on harvest delay

The berry ripening process during the two seasons considered in the study is presented in Fig 1. The immature green stage lasted from June, 10–13 to June, 24–26 for both bilberry and blueberry fruits. Berries of both species began to turn pink around June, 24–26 and this phase lasted until June, 8–10 in 2013 for both bilberry and blueberry, while in 2014 this phase was shorter as bilberries and blueberries started to turn purple-blue between June, 29 and July, 3.

Bilberries started to turn purple-blue around July 8, in 2013 and were completely ripe on July, 25 while for blueberry ripening was delayed for a couple of days, starting around July, 10 and lasting until July 28, 2013. For blueberries grown under sunlight and white net, 100% of berries were ripe after 8 weeks from the experimental setup (July, 16) while under blue and red nets the same percentage was reached one week later (July, 28). Under black net, the ripening was delayed for about 20 days, as after 9 weeks from the experiment setup (July, 28) only 50% of fruits had reached maturity.

In 2014, berry maturation was anticipated of about 10 days, starting around June, 29 and finishing between July, 3–10 for bilberry; for blueberry it started around July, 3 and was completed between July, 9–18. During the season 2014, the ripening was fast and homogeneous as all blueberries ripened at the same time under blue, red and white nets, and under sunlight. Under the black net, only 50% of blueberries were ripe at the time when other plants reached full ripeness.

### Temperature flow during berry ripening

The temperature of the air in open-field differed in day and night records between the two growing seasons. Considering the period between the experimental net setup (June, 10–13) and the time of harvesting (July, 18–28), relevant differences in temperature flows were recorded during green, pink and purple-blue stages of fruit development (Fig 1).

Days during green stage of fruit development in 2013 were characterized by an average of 28.5°C in maximum daily temperatures and of 12.3°C in minimum temperatures, with daily excursions of 14°C. Lower temperatures were recorded in 2014, between June, 15 and June, 21 with a difference in maximum daily temperatures ranging between 4 to 9.4°C lower than in 2013. Similarly, night temperatures were 2.7–6.4°C lower, decreasing, therefore, daily excursions compared to 2013.

The period of pink colored fruits, common to both species, was characterized by similar temperatures during daytime (maximum 20–25°C, daily excursion 12°C), however, lower night temperatures were recorded between June, 26 and July 1, 2013, with a difference in minimum temperatures ranging between 3–10°C lower than in 2014.

Days relative to the last ripening phase in 2013 were characterized by progressively increasing maximum daily temperatures from 26–28°C to 30–34°C, and night temperatures between 14 and 17°C. In 2014, daily temperatures fluctuated between 20–28°C, and night temperatures between 9–16°C (Fig 1).

### Temperature and humidity under photo-selective shading nets during berry development

During the fruit ripening, the temperature of the air under the nets was recorded every hour continuously for one week. Data loggers revealed that nets modified the air temperature surrounding the crop with cyclic patterns during the day (Fig 2A). White nets increased the temperature of the air over the crop during the night hours and in the morning (until 1pm). The average increase induced by the white net was ranging between 0.7°C during the night hours and 1°C between 8am and 1pm. It followed a marked and progressive decrease in the air temperature during the warmest hours of the day (1pm-5pm) when temperature was decreased up to 1°C compared to open-field temperature (sun). Temperature increased again during night hours (after 9pm; Fig 2A). Temperature under red net was following the temperatures in open-field, except during sunlight exposure hours (9am-4pm) when temperature was lowered up to 0.5°C (Fig 2A).

Blue and black net decreased the air temperature 0.1–0.5°C during the night hours. However, during day-time temperature increased up to 1.5°C during the sunlight exposure hours (10am-4pm) and decreased during following hours (4pm-12pm) under blue net. On the contrary, under black net, despite an increase in temperature of 0.8°C at 10am, a sharp and linear decrease followed (up to 2.5°C) until 1pm. After that, temperature began to increase but still maintained lower than in open-air during sunlight exposure hours until 6pm when temperature instead increased of up to 1°C until 10pm in the evening (Fig 2A).

Relative humidity (RH) was measured contemporarily to temperature during the fruit ripening. Data loggers revealed that RH changed cyclically during the day, increasing during the night hours while decreasing during the sunlight exposed hours. The highest RH (100%) was recorded at dawn (8am). Later, RH initially decreased sharply and linearly in the morning (before noon) and decreased in the afternoon, reaching the minimum (30%) at 5pm. During sunset RH started to increase over linearly (Fig 2B). RH was also modified under nets; RH was 10% lower under blue and white nets during the night hours (9pm-8am) compared to black and red nets, under which RH was not modified. Under blue net, RH was also lower during the day hours (Fig 2B).

### Solar radiation, light spectrum and photosynthetic activity under photo-selective shading nets during berry development

The colored photo-selective nets modified the intensity and the spectrum of the light reaching the plants (S1 Fig and S2 Table). Light measurements confirmed that in clear sky days, blue, red and white nets, with suitable number of layers, screened 25% of sunlight radiation, while under black net shading reached 90%.

**Fig 3:**
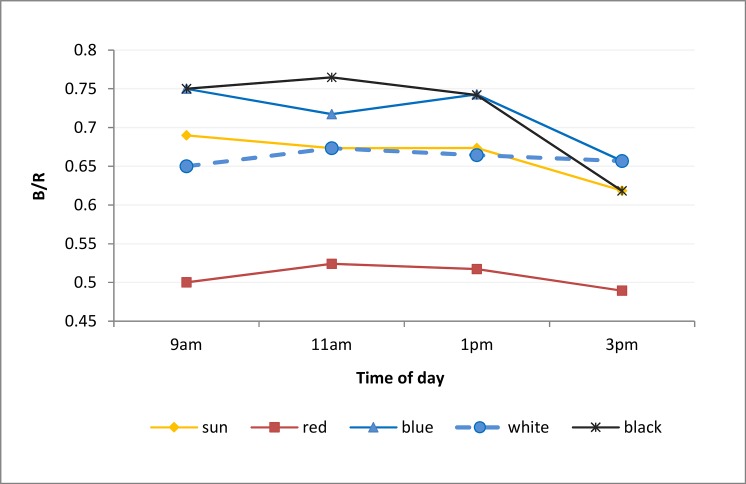
Ratios of blue/red (B/R) components of visible light spectrum were measured during daytime under clear sky day (June 30, 2013).

The ability of the nets to modify the light spectrum of the sun was measured through the UVA and UVB components and the ratio of B/R and R/FR components, as the B, R and FR components varied along the day under the different treatments (Fig 3 and S2 Table). The UVA bands generally counted for the 1–3% of the total sunlight radiation, while the UVB bands were not detected by the instrument. Under the white net the UVA component was increased compared to open-field conditions, while they were completely screened under the black net. Blue and red nets screened the UVA radiation, with major effect by the red net under which also cut the blue bands from the light spectra (S1 Fig).

The B/R ratio of sunlight radiation remained constant during the daylight hours, with an average value of 0.66. The white net showed similar trend of values to open-field conditions, whereas under the black net the B/R ratio was markedly increased up to 0.75. The ratio under the black net was generally higher during the morning while decreasing in the afternoon. The blue net showed values and trends similar to black one. Moreover, under the blue net the B/R value appeared to cycle, being up at 9am and 1pm but going down at 11am and 3pm. Under the red net, most of the blue wavelengths were removed from the light spectra (S1 Fig) and the B/R value was the lowest among all the light treatments, ranging between 0.49 and 0.52. It is interesting to observe that the B/R values varied markedly during the morning hours, while they all reached the same values (except for the red net) at 3pm (Fig 3 and S2 Table). The R/FR ratio instead was constant throughout the light hours of the day and values were similar for all the different nets, with average values of 1.23±0.23. A single exception was recorded under black net in the early morning (9am), when the R/FR ratio was 2- to 3- folds higher than during the rest of day. Green light was reduced according to the shading percentage of the net applied; it was markedly reduced under the black net during the morning hours (S2 Table).

The photosynthetic rate was similar at the top and middle part of the plant, whereas it was reduced in the bottom leaves, and was in line with levels of PAR. An exception was constituted by the black net, under which the PAR was reduced at the top and middle plant but photosynthetic activity was maintained as highest among the treatments. Under white net PAR was similar to the open-field conditions and in bottom leaves it was even higher and corresponded to an increase in the photosynthesis rate (S2 Fig).

### Berry size, weight and TSSC

The use of colored photo-selective nets influenced the final size of blueberries at ripeness. The red, blue and white colored nets increased significantly the size and weight of the single berries compared to berries grown in open-field. Under the black net, blueberries reached sizes comparable with the other light treatments, except that weight of berries was lowest during the first season of the experiment. However, during both seasons the TSSC was lowest in berries grown under black nets with average values between 11.2–11.5°Brix (S3 Table).

On the contrary, the presence of the nets over the bilberry crop did not affect remarkably the soluble solid content of the berries, which ranged between 8.1–14.6°Brix, with the highest values recorded in bilberries grown under sunlight (S3 Table).

### Anthocyanin profiles of wild vs. cultivated *Vaccinium* berries

The two *Vaccinium* species considered in this study, presented different AC profiles. In wild bilberry, 36 ACs were identified, which included the arabinosides (ara), galactosides (gal) and glucosides (glu) of delphinidins (Dp), cyanidins (Cy), peonidins (Pn), petunidins (Pt) and malvidins (Mv), and acylated forms of these (which included acetylated and coumarylated ACs). Three new acetylated compounds were detected: Dp acetyl 3 gal, Pn 3 acetyl 3 gal and Pt acetyl 3 gal (S1 Table). In small amounts, also Cy 3 sambubioside and pelargonidins (Pg) were identified (Table 2). Bilberry showed the typical anthocyanidin profile of bilberries from Southern Europe, with the highest proportion of ACs due to accumulation of Dp, followed by Cy and Mv, and in lowest amounts Pn and Pt [28–31].

**Table 2:** List of anthocyanins (AC) identified in wild bilberries grown under different photo-selective nets during 2013 and 2014.

	Year 2013				Year 2014				Statistics		
AC	Sun	Red	Blue	Black	Sun	Red	Blue	Black	Year	Net	Year*Net
Dp 3 glu	135.5±48.5	135.8±24.7	211.0±19.8	145.5±59.4	183.0±53.0	145.0±40.0	138.0±12.0	230.0±79.0			
Dp 3 gal	143.0±47.8	118.4±21.6	190.3±12.6	178.2±38.6	140.0±32.0	134.0±40.0	119.0±10.0	171.0±26.0			*
Dp 3 ara	139.4±50.1	144.7±31.1	225.2±9.3	190.0±21.5	182.0±45.0	177.0±39.0	170.0±13.0	230.0±25.0			
Dp acetyl 3 glu	0.3±0.3	0.3±0.2	0.5±0.4	0.3 ±0.3	n.d	n.d	n.d	n.d.	***		***
Dp acetyl 3 gal	0.3±0.2	0.3±0.0	0.4±0.1	0.5±0.5	n.d	n.d	n.d	n.d.	***	*	***
Dp coum 3 glu	1.8±1.5	2.1±0.1	3.1±1.2	2.9±2.0	0.2±0.1	0.1±0.1	0.2±0.0	0.4±0.1	***		**
Dp coum 3 gal	2.3±2.1	2.5±0.7	4.2±0.6	3.7±2.4	0.3±0.2	0.2±0.0	0.2±0.1	0.3±0.3	***		*
**Total Dp**	**621.5±179.0**	**589.3±92.8**	**893.9±96.5**	**799.9±182.5**	**505.8±119.7**	**455.8±118.7**	**426.9±33.6**	**630.7±130.6**		*	*
Cy 3 glu	39.1±6.8	32.3±6.8	36.1±10.1	38.6±7.1	62.0±20.0	51.0±22.0	72.0±5.0	77.0±6.0	***		**
Cy 3 gal	47.3±10.0	34.9±10.0	34.3±7.4	41.2±11.5	51.0±17.0	47.0±20.0	68.0±3.0	63.0±13.0	*		*
Cy 3 ara	50.4±8.6	63.5±21.2	46.7±11.5	58.1 ±20.2	16.0±5.0	16.0±7.0	21.0±1.0	20.0±4.0	**		**
Cy acetyl 3 glu	0.5±0.3	0.5±0.2	0.6±0.4	0.8±0.4	0.2±0.2	0.2±0.2	0.2±0.2	0.2±0.2	*		
Cy acetyl 3 gal	n.d.	n.d	n.d.	n.d.	0.2±0.1	0.2±0.1	0.3±0.1	0.2±0.1	***		**
Cy coum 3 glu	1.7±0.9	1.7±0.5	1.7±0.7	2.3±1.0	0.8±0.4	0.6±0.4	0.5±0.5	0.5±0.5			
Cy coum 3 gal	1.9±0.8	1.7±0.6	1.6±0.5	2.3±1.0	0.6±0.4	0.6±0.4	0.9±0.1	1.0±0.15	*		
Cy 3 samb	0.1±0.1	0.1±0.0	0.1±0.1	0.1±0.0	0.2±0.1	0.2±0.1	0.2±0.1	0.1±0.1			
**Total Cy**	**141.0±23.1**	**134.7±10.4**	**121.2±26.7**	**143.4±36.0**	**131.3±42.1**	**114.7±49.3**	**162.3±8.5**	**161.1±24.1**	*		**
Pt 3 glu	14.9±4.8	15.1±2.7	24.3±4.3	22.6±5.0	40.0±10.0	33.0±6.0	31.0±2.0	48.0±0.1		*	
Pt 3 gal	11.8±3.4	9.8±1.5	15.1±1.1	17.6±5.6	20.0±5.0	20.0±4.0	18.0±2.0	23.0±1.0		*	
Pt 3 ara	11.1±3.3	11.5±2.3	16.0±2.1	15.5±2.2	15.0±4.0	16.0±1.0	15.0±1.0	18.0±1.0		**	
Pt acetyl 3 glu	0.1±0.1	0.1±0.0	0.2±0.0	0.2±0.2	0.1±0.1	0.1±0.02	0.1±0.04	0.1±0.0	*		*
Pt acetyl 3 gal	0.0±0.0	0.1±0.0	0.1±0.0	0.1±0.1	n.d.	n.d.	n.d.	n.d.	***	*	***
**Total Pt**	**37.9±10.8**	**36.6±5.6**	**55.8±7.0**	**56.1±12.4**	**74.8±18.1**	**68.8±11.6**	**63.8±4.8**	**88.0±11.3**		*	
Pn 3 glu	41.4±11.1	30.6±16.6	53.8±0.8	48.3±9.6	32.0±11.0	30.0±14.0	42.0±3.0	40.0±4.0			*
Pn 3 gal	19.0±5.9	19.8±11.8	16.9±5.0	18.6±3.6	7.0±2.0	7.0±3.0	9.0±0.8	8.0±2.0	***		***
Pn 3 ara	6.2±1.5	5.6±1.0	5.7±1.9	6.7±1.0	4.0±3.0	6.0±3.0	8.0±0.7	3.0±2.5	**		*
Pn acetyl 3 glu	0.4±0.4	0.5±0.2	0.6±0.4	1.0±0.6	0.0±0.0	0.1±0.0	0.1±0.0	0.1±0.0	***		***
Pn acetyl 3 gal	0.2±0.2	0.3±0.2	0.2±0.1	0.4±0.2	n.d.	n.d.	n.d.	n.d.	***		***
Pn coum 3 glu	0.4±0.3	0.5±0.2	0.6±0.3	0.8±0.5	0.1±0.0	0.1±0.1	0.2±0.1	0.2±0.0	***		***
Pn coum 3 gal	0.2±0.1	0.2±.1	0.2±0.1	0.3±0.1	0.0±0.0	0.1±0.0	0.1±0.0	0.0±0.0	***		*
**Total Pn**	**67.9±17.8**	**57.5±22.4**	**77.8±7.9**	**76.1±14.0**	**38.5±12.5**	**36.5±16.7**	**51.5±3.2**	**47.8±6.4**			
Mv 3 glu	22.1±6.4	23.2±2.2	38.3±12.4	33.5±10.8	53.0±13.0	49.0±5.0	49.0±7.0	66.0±4.0	***		
Mv 3 gal	9.3±3.0	8.0±1.3	13.4±2.8	19.6±9.5	15.0±5.0	18.0±4.0	16.0±3.0	16.0±2.0	*		
Mv 3 ara	12.5±3.5	13.3±1.9	18.3±5.6	26.8±12.4	17.0±5.0	20.0±3.0	21.0±3.0	20.0±6.0			*
Mv acetyl 3 glu	0.1±0.1	0.2±0.1	0.4±0.2	0.4±0.3	0	0	0	0	***	**	***
Mv acetyl 3 gal	0.1±0.1	0.1±0.0	0.2±0.1	0.2±0.2	n.d.	n.d.	n.d.	n.d.	***	*	***
Mv coum 3 glu	12.0±10.0	17.0±3.0	35.0±18.0	32.0±26.0	0.6±0.3	0.5±0.1	0.7±0.1	0.9±0.0	***	**	***
Mv coum 3 gal	6.0±4.0	8.0±1.0	16.0±8.0	15.0±14.0	0.3±0.1	0.3±0.1	0.3±0.1	0.4±0.1	***	**	***
**Total Mv**	**62.3±21.8**	**69.8±7.7**	**120.9±45.8**	**127.6±45.6**	**84.8±21.6**	**86.7±9.4**	**86.7±12.8**	**101.7±5.0**	**		**
Pg 3 glu	0.4±0.1	0.4±0.0	0.5±0.1	0.3±0.3	0.4±0.2	0.3±0.1	0.4±0.0	0.5±0.1			*
Pg 3 gal	0.2±0.1	0.2±0.1	0.2±0.0	0.2±0.2	0.2±0.1	0.1±0.1	0.2±0.1	0.3±0.0			
**Total Pg**	0.7±0.2	0.6±0.1	0.7±0.1	0.6±0.5	0.6±0.2	0.4±0.2	0.6±0.1	0.7±0.0		***	***
**Total AC**	**931.3±219.7**	**888.4±84.0**	**1270.3±180.3**	**1203.7±259.8**	**835.1±159.5**	**762.9±88.5**	**791.7±54.5**	**1030.0±106.5**	*	*	

The accumulation of the compound was significantly affected by the parameter, as indicated by two-way ANOVA test

*** p<0.001

** p<0.01

* p<0.05.

n.d. = not detected.

In blueberry, 15 AC compounds were identified, which included the ara, gal and glu forms of Dp, Cy, Pn, Pt and Mv, but no acylated forms were identified. The profile of anthocyanidins was typical for blueberry, with Dp and Mv as predominant anthocyanidins, followed by Pt and in lowest proportion Cy and Pn [30, 32].

Significant differences were also recorded within the same species during the two growing seasons. In bilberry, a lower content of total AC, and in general for all classes of anthocyanidins was recorded in 2014 (Table 2). Acylated compounds were the most affected (p<0.05), as these compounds were synthetized in lower amount or not detected in fruits collected during 2014 (Table 2). However, Cy acetyl 3 gal was produced in 2014, which was not detected in 2013. The more abundant ACs (non acylated forms) were not affected except the group of Cy (Cy 3 glu, Cy 3 gal, Cy 3 ara) and Mv (Mv 3 glu, Mv 3 gal) which were higher in 2014 (p<0.05). On the contrary, Pn’s (Pn 3 gal, Pn 3 ara) were higher in 2013 (p<0.01).

In blueberry (Table 3), the year significantly affected the accumulation of the total ACs in blueberries (p<0.001) which produced higher quantities of ACs in 2013, due to a major effect of the year on the accumulation of Dp and Pn (p<0.01). Also Pt and Mv contributed to the higher amount of AC in 2013 (p<0.05) but in minor part, as only Pt 3 glu, Pt 3 ara and Mv 3 glu were produced in lower amount in 2014.

**Table 3:** List of anthocyanins (AC) identified in blueberries grown under different photo-selective nets during 2013 and 2014.

	Year 2013					Year 2014					Statistics		
AC	Sun	Red	Blue	White	Black	Sun	Red	Blue	White	Black	Year	Net	Year*Net
Dp 3 glu	5.3±0.7	3.4±1.3	3.5±0.7	2.9±1.3	1.5±0.7	3.0±0.7	2.6±0.5	2.3±0.4	2.3±0.6	1.6±0.2	**		
Dp 3 gal	114.0±13.6	76.1±15.6	83.2±12.1	66.5±27.5	36.2±13.4	69.0±11.1	65.4±15.3	52.8±9.0	58.7±14.9	43.3±1.5	**		*
Dp 3 ara	113.6±12.3	77.4±13.0	83.6±18.0	68.5±22.1	38.7±11.4	56.4±6.7	58.3±8.7	52.8±9.0	56.0±10.9	43.5±2.0	***	**	*
**Total Dp**	**233.6±26.6**	**157.1±29.7**	**170.8±31.0**	**138.2±51.1**	**76.6±25.4**	**128.3±18.4**	**126.3±24.4**	**107.9±18.3**	**117.0±26.4**	**88.3±3.6**	**	*	
Cy 3 glu	0.3±0.0	0.2±0.1	0.2±0.1	0.2±0.1	0.1±0.1	0.3±0.0	0.2±0.1	0.2±0.0	0.2±0.1	0.1±0.0		*	*
Cy 3 gal	4.0±0.5	3.0±1.6	2.7±0.5	2.2±1.0	2.1±0.4	6.0±1.0	4.8±1.9	4.1±0.8	6.0±1.0	3.1±0.3		*	**
Cy 3 ara	3.2±0.4	2.4±1.1	2.3±0.4	1.9±0.8	1.6±0.3	1.4±0.2	1.2±0.4	1.1±0.3	1.0±0.3	0.8±0.1	***		**
**Total Cy**	**8.5±1.6**	**5.9±2.6**	**5.7±1.5**	**4.3±1.9**	**3.8±0.8**	**7.7±1.2**	**6.3±2.3**	**5.4±1.1**	**7.1±0.7**	**4.1±0.4**		**	*
Pt 3 glu	0.8±0.1	0.5±0.2	0.6±0.1	0.5±0.2	0.4±0.0	0.7±0.1	0.8±0.1	0.7±0.1	0.6±0.1	0.5±0.1	*		
Pt 3 gal	15.9±2.3	11.6±1.7	12.0±2.0	9.7±3.6	7.2±0.8	16.2±2.0	14.2±3.3	14.0±2.0	15.5±2.5	9.5±3.3			
Pt 3 ara	12.8±1.5	9.5 ±1.4	9.7±1.6	8.2±3.0	6.3±0.5	9.5±1.0	10.0±1.1	9.9±1.2	9.5±1.7	7.8±0.5	**		
**Total Pt**	**29.6±3.8**	**21.6±3.2**	**22.3±3.7**	**18.3±6.8**	**13.9±1.2**	**26.4±3.0**	**24.9±3.5**	**24.6±3.3**	**25.6±2.3**	**17.8±3.9**	*		
Pn 3 glu	0.5±0.1	0.5±0.3	0.4±0.1	0.3±0.1	0.4±0.1	0.2±0.0	0.2±0.1	0.2±0.0	0.1±0.0	0.1±0.0	**		**
Pn 3 gal	6.5±0.5	5.7±2.0	5.1±1.5	3.6±1.3	4.7±0.5	2.2±0.3	2.1±0.6	2.0±0.3	1.8±0.4	1.5±0.1	**		**
Pn 3 ara	3.4±0.3	2.7±0.4	2.8±0.7	2.2±0.6	2.3±0.2	0.3±0.1	0.3±0.1	0.4±0.1	0.3±0.1	0.3±0.1	***	*	**
**Total Pn**	**10.4±0.8**	**8.9±2.7**	**8.3±2.2**	**6.2±2.1**	**7.4±0.6**	**2.7±0.4**	**2.6±0.8**	**2.5±0.4**	**2.2±0.6**	**1.9±0.2**	***		**
Mv 3 glu	3.7±0.6	2.1±0.3	2.3±0.4	2.0±0.7	1.5±0.2	2.0±0.1	2.1±0.3	2.1±0.4	1.9±0.4	1.6±0.1	**	***	
Mv 3 gal	34.8±2.6	27.3±2.6	27.6±5.5	22.5±5.9	20.1±1.7	32.7±1.6	36.5±3.8	36.8±4.0	34.5±6.0	29.2±2.0			
Mv 3 ara	27.3±2.6	22.3±0.8	23.3±4.7	18.9±3.9	17.8±1.0	17.9±0.9	22.0±1.9	23.7±2.7	22.3±3.3	20.4±1.6		*	
**Total Mv**	**70.0±4.8**	**51.7±3.5**	**53.4±10.6**	**43.4±10.5**	**39.4±2.1**	**52.6±2.5**	**60.5±5.9**	**62.6±7.1**	**58.7±9.7**	**51.2±3.6**	*		
**Total AC**	**346.9±64.0**	**244.8±23.0**	**259.8±47.4**	**210.4±42.1**	**141.1±27.3**	**217.7±25.2**	**220.6±30.7**	**207.3±20.2**	**210.6±36.5**	**163.4±8.8**	***	*	*

The accumulation of the compound was significantly affected by the parameter, as indicated by two-way ANOVA test

*** p<0.001

** p<0.01

* p<0.05.

n.d. = not detected.

### Accumulation of anthocyanidins in berries in response to microclimate under photo-selective nets

The microclimate under the photo-selective nets affected significantly the accumulation of ACs in both species (bilberry and blueberry) although in a different way. Bilberry plants did not survive under the white net, which therefore was not considered for this species in the study. The highest amount of ACs was detected in bilberries grown under the black net both in 2013 and 2014, which was significantly higher (p<0.01) than in bilberries grown under red net and in open-field—under direct sun. Under the blue net, ACs accumulated at the same level as under the black net in 2013, due to a significant increase in the accumulation of Dps and Mvs over red net and sun-grown bilberries (Table 2). In 2014, under the blue net the level of ACs was instead similar to bilberries ripened under the red net and direct sun, as Dps were produced in lower amount compared to the previous year.

In blueberry, the accumulation of ACs in response to microclimate presented opposite trend, as it was significantly decreased under the black net, compared to all other treatments in both years (p<0.01; Table 3). The decrease was accompanied by a significant decrease in Dp (p<0.01), Cy and Pn (p<0.05). In blueberries ripened under the blue, red and white nets, the content of total ACs was similar, and no differences in anthocyanidin classes were found in both seasons (Table 3). Significant differences were recorded in 2013 between blueberries grown in open-field compared to all other treatments (p<0.05), as blueberries grown under direct sunlight produced the highest amount of ACs (347±64 mg/100 g FW) accompanied by highest production of all classes of anthocyanidins (Table 3). The trend was not repeated in 2014, as blueberries under sunlight accumulated the same amount of ACs as under colored nets (Table 3).

## Discussion

Photo-selective netting refers to covering crops by nets which have the capacity to selectively filter the intercepted solar radiation, in addition to their protective function. The technology is based on plastic net products into which various chromophores and light dispersive and reflective elements are introduced during manufacturing [33]. This design enables nets to screen specific spectral bands of the solar radiation, such as under blue and red nets in the present study (S1 Fig) and/or transform direct light into scattered light–in the present study under white net. The spectral manipulation intends to specifically promote desired physiological responses, which are light-regulated, while the scattering improves the penetration of the modified light into the inner plant canopy (S2B Fig, [33]). In the present study, nets showed also ability to affect the temperature and the relative humidity of the canopy during day and night (Fig 2), creating therefore a particular microclimate, which was a specific combination of light intensity and spectrum, temperature and RH, characteristic for each net tested.

In many fruits, the accumulation of ACs is developmentally regulated as it typically occurs during the fruit ripening. However during the past few years, numerous studies have shown that light intensity, light quality and temperature may induce quantitative and/or qualitative changes in the AC profile of apples, grape berries, strawberries and bilberries [34]. In the present study, the use of the nets highlighted a different responsiveness of the examined *Vaccinium* species to different combinations of light and temperature conditions, although bilberry and blueberry are both considered to be shade-adapted plants. Our results showed that under the black net, where the microclimate was characterized by low light intensity and temperatures lowered of 0.5–2.5°C compared to open-field conditions, bilberries accumulated the highest content of ACs in both seasons, whereas blueberries accumulated the lowest (Tables 2 and 3). On the contrary, higher light intensity favored the production of high amounts of ACs in blueberries but not bilberries (Tables 2 and 3).

Earlier studies indicate that the optimal light conditions for growth and development of bilberries are under 65% shading level, when PAR is about 35% of solar radiation [35]. In the present study, the plants under the white net did not survive. Whether this was related to the enhanced UVA radiation under the net (compared to open-field control; S2 Table), remains to be further evaluated. However, our results suggest that an excessive light stress may inhibit the accumulation of ACs in bilberry fruits. The highest AC contents were reached under the black and blue nets, which presented similar temperature trend–i.e. lowered temperatures during the night, and a marked difference between maximum and minimum temperatures during daily hours (Fig 2A). In addition, despite that the light intensity was not comparable, both nets transmitted higher proportions in the blue bands of the light spectra (Fig 3). In bilberries under these light-temperature combinations, the anthocyanidin composition of Dps, Pts and Mvs was significantly increased, whereas Cys and Pns were not affected (Table 2).

In our previous study on the effect of light quality on AC accumulation in bilberry, blue bands of the light spectra increased the accumulation of the more hydroxylated ACs Dps, Pts and Mvs, but not Cys and Pns [20]. Moreover, the production of Dps was stimulated by lower temperatures of 12°C (compared to higher of 18°C) in temperature controlled conditions [36]. These results indicate that the specific combination of light and temperature conditions have a role in regulation of AC composition of bilberries. This aspect can be further supported by the present results, as under the red net where temperatures were similar to open-field conditions throughout the day (Fig 2A) and where the blue bands were removed from the light spectra (Figs 3 and S1), the content and profile of ACs was most similar to bilberries grown in open-field conditions (Table 2). These results are also in accordance with Kondo et al. [13] who found that AC accumulation in grape berries was more stimulated by blue light than red light. Green light supplementation was found to inhibit the blue light-stimulation of AC accumulation in *Arabidopsis* seedlings [37, 38], but had relatively minor effects in grape berries [39]. The present results do not show evident effects of the green light component on AC accumulation in *Vaccinium* berries, but this aspect needs further evaluation.

Different results were observed for blueberries, which showed the best production–in terms of berry size (S3 Table)–in conditions of 25% shading or in open-field conditions. In opposite way to bilberry, excessive light shading conditions (90%) were not favorable for blueberry production as maturation was delayed for at least two weeks. This is in line with the results on cv. Elliott cultivated in Chile, for which it was showed that blueberry fruit yield and soluble solids had a positive relationship with % PAR [25]. Indeed, despite blueberry plants kept high photosynthetic rates under the black net (S2 Fig), the soluble solid content as well as ACs were significantly decreased in cv Brigitta Blue compared to berries grown under sunlight conditions (Tables 3 and S2). In blueberries ACs accumulate only in the berry skin which is found to increase total AC content as the surface area-to-volume of the berry increases [40]. In the present study, the effect of nets on AC accumulation was positively correlated with the size of blueberries, as indicated by the Pearson’s analyses (S4 Table). The colored nets (blue, red and white) stimulated the development of the berries which was shown as significant increase in berry size and weight compared to berries grown under sunlight (S3 Table). The increase in the berry volume also increased the berry water content and therefore the final AC content in blueberries ripened under the colored nets was lower than in berries grown under sunlight (Table 3). According to Lobos et al. [24], photo-selective nets decrease light intensity and temperature over highbush blueberry plants, and enhance their photosynthesis efficiency. The impact of light quality seems to be less strong than its amount, where in general up to 50% PAR shows similar responses with open-field conditions.

Light conditions interacted with environmental temperatures which varied during the two growing season and had a strong effect on the accumulation of ACs in both species. The higher AC yield was recorded in 2013, when berry development occurred slower than in 2014 and pink and purple-blue ripening phases (Fig 1) indicating AC accumulation [5] to take longer time. The slower ripening in 2013 could be a consequence of the low night temperatures recorded between June, 26 and July, 1 (8–11°C) which delayed the harvesting compared to season 2014, but increased the AC content in line with results on grape [41]. During ripening phases, however, berries encountered generally the optimum temperatures for blueberry fruit set, size and ripening, which are 20–26°C during the day and 16°C at night [7]. For bilberry, our previous temperature controlled study showed that plants grown at 18°C produced higher yields of fruits with higher amounts of ACs compared to lower temperature at 12°C [36]. The present study showed that bilberry plants tolerated well temperatures higher than 20°C, and the berries reached full ripeness even in the presence of temperatures between 26–30°C in 2013. Longer exposures of bilberries to temperatures between 26–30°C during the last phase of ripening in 2013, apparently further modified their AC profile resulting in higher concentrations of acylated forms of AC (Table 2), similarly with findings in grape berries (cv. Merlot) [42].

Our results suggest that the use of colored photo-selective nets may be a complementary agricultural practice for cultivation of *Vaccinium* species. For instance, black netting could be employed in the emerging attempts for semi-cultivation of bilberry [43] to produce berries of high nutritional value and to increase the commercial value of the crop which could be exploited in industry lines interested. However, in the present study, the high shading percentage affected negatively the flower induction on the second year production. Thus the nets of black or blue color with lower shading percentage should be preferred for cultivation purposes.

In general, further research is needed to analyze the effect of the light spectra modifications to other nutritional properties of berries, and to optimize both the production as well as the nutritional value of the fruits. However, results give also exciting prospects for further gene x environment interaction studies on the regulation of the metabolic pathways in berry producing species, indicating the need to elucidate the molecular mechanisms behind the different responses of the two *Vaccinium* species to light conditions, which is clearly species-dependent.

## Supporting Information

S1 Fig Comparison of visible light spectra were recorded under direct sun and under blue, white, red and black nets. Spectra were measured at noon in a clear sky day (June 30, 2013).
(TIF)

S2 Fig Photosynthetic activity (a) and PAR (b) were measured on leaves at three different heights (top, middle and bottom) of the blueberry bushes under different light treatments. Color bars: blue: top of the plant, red: middle, green: bottom.
(TIF)

S1 Table UPLC-MS/MS data for anthocyanin quantification. In case of two MRM transitions for a given compound, the first was used as quantifier and the second as qualifier. RT = retention time, CV = cone voltage, CE = collision energy.
(DOCX)

S2 Table Light measurements taken at the top of the canopy every 2^nd^ hour between 9am and 3pm during the ripening period of berries (June 30, 2013). Measurements were done on clear sky sunny day. The quality of light reaching the plants was identified measuring the photosynthetic active radiation (PAR; Photons/cm²/s) values between 200–850 nm for full light spectra, 280–320 nm for UVB, 280–380 nm for UVA, 380–495 nm for blue (B), 495–590 for green (G), 590–710 nm for red (R) and 710–750 nm for far-red (FR) components of the sunlight spectra. The ratios of B/R and R/FR were also calculated.
(DOCX)

S3 Table Pomological analyses (single berry weight, diameter and height), total soluble solid content (TSSC) and acidity of berries at harvesting. (DOCX)

S4 Table Correlation between pomological analysis and the anthocyanin content in blueberries (Pearson’s analysis). Correlations are significant when p<0.05 and are marked with asterisk (*).
(DOCX)
