# Supplementary figures and images for: Modification of Sunlight Radiation through Colored Photo-Selective Nets Affects Anthocyanin Profile in Vaccinium spp. Berries
Source: PLoS One. 2015 Aug 19;10(8):e0135935. doi: 10.1371/journal.pone.0135935 (PMC4545418; doi:10.1371/journal.pone.0135935)

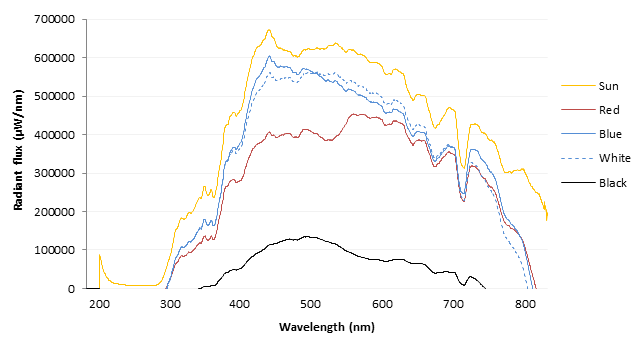

Supplement: S1 Fig — Spectra were measured at noon in a clear sky day (June 30, 2013). (TIF) [file pone.0135935.s001.tif]

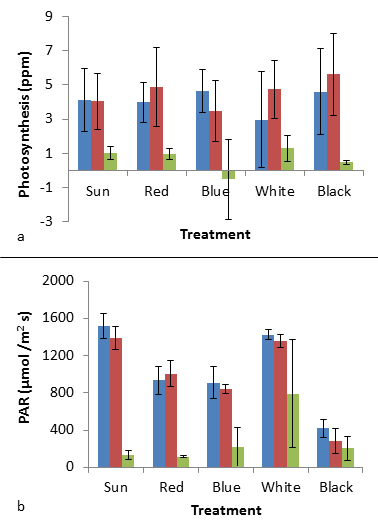

Supplement: S2 Fig — Color bars: blue: top of the plant, red: middle, green: bottom. (TIF) [file pone.0135935.s002.TIF]
