# Supplementary material for: Modification of Sunlight Radiation through Colored Photo-Selective Nets Affects Anthocyanin Profile in Vaccinium spp. Berries
Source: PLoS One. 2015 Aug 19;10(8):e0135935. doi: 10.1371/journal.pone.0135935 (PMC4545418; doi:10.1371/journal.pone.0135935)
Supplement: S1 Table — In case of two MRM transitions for a given compound, the first was used as quantifier and the second as qualifier. RT = retention time, CV = cone voltage, CE = collision energy. (DOCX) [file pone.0135935.s003.docx]

| **Peak** | **Identification** | **RT** | **MRM transitions** | **CV** | **CE** | **Quantified by** |
| --- | --- | --- | --- | --- | --- | --- |
| 34 | Dp acetyl 3 gal | 3.91 | 507 → 303; 229 | 30 | 30; 50 | Mv 3 glu |
| 35 | Pn acetyl 3 gal | 4.77 | 505 → 301; 286 | 30 | 28; 50 | Mv 3 glu |
| 36 | Pt acetyl 3 gal | 4.45 | 521 → 317; 302 | 28 | 24; 46 | Mv 3 glu |
