# Supplementary material for: Modification of Sunlight Radiation through Colored Photo-Selective Nets Affects Anthocyanin Profile in Vaccinium spp. Berries
Source: PLoS One. 2015 Aug 19;10(8):e0135935. doi: 10.1371/journal.pone.0135935 (PMC4545418; doi:10.1371/journal.pone.0135935)
Supplement: S2 Table — Measurements were done on clear sky sunny day. The quality of light reaching the plants was identified measuring the photosynthetic active radiation (PAR; Photons/cm²/s) values between 200–850 nm for full light spectra, 280–320 nm for UVB, 280–380 nm for UVA, 380–495 nm for blue (B), 495–590 for green (G), 590–710 nm for red (R) and 710–750 nm for far-red (FR) components of the sunlight spectra. The ratios of B/R and R/FR were also calculated. (DOCX) [file pone.0135935.s004.docx]

| **Time of day** | **9am** | | | | | **11am** | | | | |
| --- | --- | --- | --- | --- | --- | --- | --- | --- | --- | --- |
| **Net** | **Sun** | **Red** | **Blue** | **Pearl** | **Black** | **Sun** | **Red** | **Blue** | **Pearl** | **Black** |
| **PAR** |  |  |  |  |  |  |  |  |  |  |
| full spectrum | 3,98E+16 | 2,60E+16 | 2,74E+16 | 3,20E+16 | 4,00E+15 | 6,21E+16 | 5,25E+16 | 5,55E+16 | 6,21E+16 | 8,26E+15 |
| UVB | 0 | 0 | 0 | 0 | 0 | 0 | 0 | 0 | 0 | 0 |
| UVA | 5,40E+14 | 3,98E+14 | 4,02E+14 | 6,69E+14 | 0 | 9,56E+14 | 6,68E+14 | 1,32E+15 | 1,46E+15 | 0 |
| B | 7,40E+15 | 4,26E+15 | 6,14E+15 | 6,61E+15 | 1,16E+15 | 1,01E+16 | 7,86E+15 | 1,04E+16 | 1,01E+16 | 2,47E+15 |
| G | 1,18 E+16 | 6,17 E+15 | 6,35 E+15 | 6,25 E+15 | 7,58 E+14 | 2,30 E+16 | 1,56 E+16 | 1,73 E+16 | 2,25 E+16 | 5,60E+14 |
| R | 1,07E+16 | 8,50E+15 | 8,20E+15 | 1,02E+16 | 1,54E+15 | 1,50E+16 | 1,50E+16 | 1,45E+16 | 1,50E+16 | 3,23E+15 |
| FR | 9,28E+15 | 6,74E+15 | 6,67E+15 | 8,27E+15 | 5,42E+14 | 1,30E+16 | 1,33E+16 | 1,20E+16 | 1,30E+16 | 3,12E+15 |
| B/R | 0,69 | 0,50 | 0,75 | 0,65 | 0,75 | 0,67 | 0,52 | 0,72 | 0,67 | 0,76 |
| R/FR | 1,15 | 1,26 | 1,23 | 1,23 | 2,84 | 1,15 | 1,13 | 1,21 | 1,15 | 1,04 |
| **Time of day** | **1pm** | | | | | **3pm** | | | | |
| **Net** | **Sun** | **Red** | **Blue** | **Pearl** | **Black** | **Sun** | **Red** | **Blue** | **Pearl** | **Black** |
| **PAR** |  |  |  |  |  |  |  |  |  |  |
| full spectrum | 7,76E+16 | 4,80E+16 | 5,45E+16 | 5,50E+16 | 1,14E+16 | 5,37E+16 | 4,56E+16 | 4,13E+16 | 3,91E+16 | 9,75E+15 |
| UVB | 0 | 0 | 0 | 0 | 0 | 0 | 0 | 0 | 0 | 0 |
| UVA | 1,23E+15 | 6,97E+14 | 9,88E+14 | 1,25E+15 | 0 | 7,49E+14 | 4,65E+14 | 5,56E+14 | 7,02E+15 | 0 |
| B | 1,30E+16 | 7,50E+15 | 1,04E+16 | 9,70E+15 | 2,30E+15 | 8,66E+15 | 5,53E+15 | 6,37E+15 | 6,37E+15 | 1,36E+15 |
| G | 2,79 E+16 | 1,28 E+16 | 1,70 E+16 | 1,75 E+16 | 3,36 E+15 | 1,83 E+16 | 1,73 E+16 | 1,56 E+16 | 6,80 E+15 | 3,84 E+15 |
| R | 1,93E+16 | 1,45E+16 | 1,40E+16 | 1,46E+16 | 3,10E+15 | 1,40E+16 | 1,13E+16 | 9,70E+15 | 9,70E+15 | 2,20E+15 |
| FR | 1,61E+16 | 1,25E+16 | 1,21E+16 | 1,20E+16 | 2,64E+15 | 1,20E+16 | 1,10E+16 | 9,52E+15 | 9,21E+15 | 2,35E+15 |
| B/R | 0,67 | 0,52 | 0,74 | 0,66 | 0,74 | 0,62 | 0,49 | 0,66 | 0,66 | 0,62 |
| R/FR | 1,20 | 1,16 | 1,16 | 1,22 | 1,17 | 1,17 | 1,03 | 1,02 | 1,05 | 0,94 |
