# Supplementary material for: Modification of Sunlight Radiation through Colored Photo-Selective Nets Affects Anthocyanin Profile in Vaccinium spp. Berries
Source: PLoS One. 2015 Aug 19;10(8):e0135935. doi: 10.1371/journal.pone.0135935 (PMC4545418; doi:10.1371/journal.pone.0135935)
Supplement: S3 Table — (DOCX) [file pone.0135935.s005.docx]

| **Year** | **Species** | **Net** | **Weight (g)** | **Diameter (mm)** | **Height (mm)** | **TSSC (°Brix)** | **Acidity (meq/100g)** |
| --- | --- | --- | --- | --- | --- | --- | --- |
| 2013 | Blueberry | Sun | 2.1 ± 0.5 | 13.5 ± 1.1 | 10.3 ± 0.8 | 12.7 ± 0.7 | 22.6 ± 5.4 |
|  |  | Red | 2.2 ± 0.3 | 15.4 ± 1.2 | 11.0 ± 0.8 | 11.8 ± 1.2 | 22.3 ± 5.4 |
|  |  | Blue | 2.4 ± 0.4 | 14.6 ± 1.9 | 11.1 ± 1.2 | 12.9 ± 1.2 | 17.9 ± 3.7 |
|  |  | White | 2.5 ± 0.4 | 15.3 ± 1.4 | 11.6 ± 1.0 | 12.0 ± 1.5 | 20.8 ± 6.2 |
|  |  | Black | 2.0 ± 0.4 | 14.4 ± 1.6 | 11.6 ± 1.3 | 11.5 ± 0.8 | 21.5 ± 5.6 |
| 2014 | Blueberry | Sun | 1.9 ± 0.3 | 14.5 ± 1.0 | 12.7 ± 0.7 | 13.2 ± 1.0 | 19.0 ± 1.5 |
|  |  | Red | 2.1 ± 0.5 | 15.1 ± 1.5 | 13.5 ± 0.9 | 12.1 ± 0.9 | 15.6 ± 1.9 |
|  |  | Blue | 2.0 ± 0.5 | 14.7 ± 1.4 | 13.0 ± 0.9 | 12.5 ± 1.2 | 14.2 ± 2.5 |
|  |  | White | 2.0 ± 0.3 | 14.9 ± 0.8 | 13.2 ± 0.6 | 11.7 ± 1.0 | 15.5 ± 1.0 |
|  |  | Black | 2.2 ± 0.4 | 14.7 ± 1.5 | 11.7 ± 1.0 | 11.2 ± 1.3 | 20.2 ± 3.3 |
| 2013 | Bilberry | Sun | - | - | - | 11.4 ± 2.4 | 20.8 ± 3.9 |
|  |  | Red | - | - | - | 11.1 ± 0.3 | 17.6 ± 3.9 |
|  |  | Blue | - | - | - | 9.5 ± 1.9 | 23.1 ± 0.5 |
|  |  | White | - | - | - | - | - |
|  |  | Black | - | - | - | 10.6 ± 2.5 | 21.4 ± 4.0 |
| 2014 | Bilberry | Sun | - | - | - | 11.6 ± 2.0 | 20.7 ± 0.6 |
|  |  | Red | - | - | - | 9.6 ± 1.4 | 18.7 ± 2.2 |
|  |  | Blue | - | - | - | 8.6 ± 1.5 | 19.9 ± 1.5 |
|  |  | White | - | - | - | - | - |
|  |  | Black | - | - | - | 9.6 ± 1.1 | 21.3 ± 2.7 |
