# Supplementary material for: Modification of Sunlight Radiation through Colored Photo-Selective Nets Affects Anthocyanin Profile in Vaccinium spp. Berries
Source: PLoS One. 2015 Aug 19;10(8):e0135935. doi: 10.1371/journal.pone.0135935 (PMC4545418; doi:10.1371/journal.pone.0135935)
Supplement: S4 Table — Correlations are significant when p<0.05 and are marked with asterisk (*). (DOCX) [file pone.0135935.s006.docx]

| **Anthocyanidin** | **Weight** | **Diameter** | **Height** | **TSSC (°Brix)** | **Acidity (meq/100g)** |
| --- | --- | --- | --- | --- | --- |
| Dp | 0.29 | -0.68* | -0.86* | 0.26 | 0.73 |
| Cy | -0.62* | -0.77* | -0.07* | 0.35 | 0.18 |
| Pn | 0.54 | -0.33* | -0.98* | 0.06 | 0.82 |
| Pt | -0.71* | -0.82* | -0.09* | 0.40 | -0.09* |
| Mv | -0.56* | -0.64* | -0.15* | 0.16 | -0.33* |
| Total | 0.12 | -0.78* | -0.77* | 0.29 | 0.61 |
